# Interleukin 17A promotes gallbladder cancer invasiveness via ERK/NF-κB signal pathway mediated epithelial-to-mesenchymal transition

**DOI:** 10.7150/jca.40656

**Published:** 2020-05-18

**Authors:** Kunlun Chen, Hongwei Tang, Pengfei Zhu, Jianwen Ye, Dong Liu, Yansong Pu, Lei Zhang, Wenlong Zhai

**Affiliations:** 1 Department of Hepatobiliary and Pancreatic Surgery, The First Affiliated Hospital of Zhengzhou University, Zhengzhou, Henan, 450052, China; 2 Departments of Gastroenterology, Shaanxi Provincial People's Hospital, Xi'an, Shaanxi 710068, China; 3 Departments of General Surgery, Shaanxi Provincial People's Hospital, Xi'an, Shaanxi 710068, China; 4 Hepatic Surgery Center, Institute of Hepato-Pancreato-Bililary Surgery, Tongji Hospital, Tongji Medical College, Huazhong University of Science and Technology, Wuhan, 430030, China; Kunlun Chen and Hongwei Tang contributed equally.

**Keywords:** Interleukin 17A, gallbladder cancer, epithelial-mesenchymal transition, invasiveness, ERK/ nuclear factor-κB signal pathway

## Abstract

As a pro-inflammatory cytokine, Interleukin 17A (IL-17A) plays an important role in pathology of tumor microenvironment and inflammatory diseases. In this study, we intend to investigate the role of IL-17A on the metastasis of gallbladder cancer (GBC) and related mechanisms. The serum levels of IL-17A were associated with node metastasis and advanced stage. We also found the pro-invasion effect of IL-17A on GBC cells. When treated with IL-17A, the protein level of epithelial marker E-cadherin in GBC cells was significantly down-regulated, while the protein level of the mesenchymal phenotype marker vimentin was significantly increased. IL-17A increased the expression of transcription factor slug, the phosphorylation of ERK1/2 and the nuclear translocation of NF-κB/p50 and p65 in a concentration-dependent manner. Pretreatment of cells with U0126 could reverse the nuclear translocation of NF-κB/p50 and p65 and EMT induced by IL-17A. IL-17A promotes gallbladder cancer invasiveness via ERK/NF-κB signal pathway mediated epithelial-to-mesenchymal transition. As a new therapeutic targets and diagnostic marker, IL-17A may play an important role in the treatment of GBC.

## Introduction

Gallbladder cancer (GBC) has the highest proportion of biliary tract tumors, which causes mass cancer-related death worldwide. The development of gallbladder cancer has been linked to various genetic and environmental factors, and chronic inflammation is the most important risk factor 1, 2. GBC is usually diagnosed at terminal stages owing to the invasive nature and contribution of the non-specific clinical symptom. The 5-year survival rates were 1%, 5%, 15%, 39% and 60% for patients with stage Ⅳ, stage Ⅲ, stage Ⅱ, stage Ⅰ, and stage 0^3^, ^4^. It is important to discover mechanisms leading to novel therapeutic targets against GBC.

As a member of IL-17 family, IL-17A was reported to have controversial roles in cancer. Our previous study suggests a promoting effect of IL-17A on tumors 5, whereas other studies suggested its protective role in cancer by enhancing immune system-mediated tumor rejection 6. Metastasis contributes to more than 90% cancer deaths 1. The role of IL-17A in the metastasis of GBC is still largely unknown.

In this study, we demonstrated that IL-17A could enhance metastatic capacity of GBC cell via promoting ERK/ NF-κB mediated EMT (epithelial-to-mesenchymal transition).

## Materials and Methods

### Reagents

Rabbit poly-clonal antibodies against E-cadherin, rabbit poly-clonal antibody against N-cadherin, rabbit mono-clonal antibody against vimentin, and goat poly-clonal antibody against Snail were purchased from Abcam. Rabbit mono-clonal antibody against Slug was purchased from Cell Signaling. Anti-NF-κB- p65, p50 and Histone H1 antibodies were ordered from Santa Cruz Biotechnology (Santa Cruz, CA, USA). DMEM (Gibco) were used. U0126 were purchased from Sigma.

### ELISA

The serums of blood samples of GBC patients were collected by centrifugation and stored at 80℃ for subsequent analysis. IL-17A levels were measured by ELISA as previously described 7. This study was approved by Ethical Committee of the First Affiliated Hospital of Zhengzhou University and in accordance with the Declaration of Helsinki. All patients signed informed consent forms.

### Cell culture

GBC-SD cell line was ordered from the Cell Bank of the Chinese Academy of Sciences (Shanghai, China). GBC-SD cells were cultured in Dulbecco's Modified Eagle's Medium (Gibco) containing fetal bovine serum (10%) and 100 μg/ml penicillin/streptomycin.

### Construction of expression plasmids and transfection

The construction of the expression plasmids and their transfection were performed as previously described 2. Briefly, we made the full-length pcDNA3.1 (Invitrogen) MEK1 vector by cloning the full-length PCR product of MEK1 with KOD® DNA polymerase (Toyobo, Osaka, Japan). We used DNA sequencing to confirm the plasmid sequences. For transient transfection experiments, cells were plated 24 h before transfection in a 6-well plate at a density of 2×10^5^ cells per well. For the transfection, we used Lipofectamine 2000 (Invitrogen) with 4.0 μg pcDNA3.1(+)-MEK1 vector or 4.0 μg pcDNA3.1(+) empty vector (as a negative control) in accordance with the manufacturer's protocol.

### Cell growth assay

The colorimetric 3-(4,5-dimethylthiazol-2-yl) 2,5-diphenyltetrazolium bromide assay (MTT) was performed to detect the effect of IL-17A on GBC cells proliferation. In brief, GBC cells (1×103 cells/well) were seeded into 96-well plates and treated with IL-17A(R&D System, Minneapolis, MN) for selected time points, and 20μl of MTT solution (5mg/ml; Sigma-Aldrich, St. Louis, MO, USA) was added and then incubated for an additional 4 h at 37˚C. The formazan crystals were dissolved in 150μl Dimethyl Sulfoxide (DMSO)(Sigma-Aldrich) and the absorbance of samples was measured at 490 nm using a micro-plate reader (Model 3550; Bio-Rad, Hercules, California, USA). The inhibitory rate of GBC cells proliferation was calculated according to the formula: (1-experimental absorbance value/control absorbance value) ×100%. Each experiment was conducted in triplicate.

### Cell migration assay

About 1x10^6^/ml GBC‑SD cells were seeded in 6‑well plates and cultured, a vertical long wound was scratched after the cells spread over the plates. GBC-SD cells were cultured with or without IL-17A (50 ng/ml) (R&D System, Minneapolis, MN) for 48 h, then image was captured. The wound closure ratio was calculated as follows: (0 h width‑48 h wound width)/0 h wound width.

### Cell invasion assay

Invasion assay was measured using 24-well BioCoat Matrigel Invasion Chambers(Becton Dicknson, Bedford, MA) as described previously^8^. After pretreated with IL-17A for 24h, cells were added into inner well and cultured for 24 h, cells that did not invade were wiped off and then cells that invaded through the matrigel was fixed with formalin, dyed with crystal violet and measured by counting the cell number under 5 microscopic vision fields.

### Western blotting analysis

Whole cell lysates were extracted with radioimmunoprecipitation assay buffer (R&D System, Minneapolis, MN) on ice for 20 min. Nuclear lysates were extracted as previously mentioned 5. Cells were suspended in 100μl of lysis buffer. After separating on 10 % SDS-polyacrylamide gel electrophoresis, proteins were transferred onto PVDF membranes. The membranes were subsequently blocked in defatted milk to reduce non-specific binding at 37˚C for 1 h and then incubated with different antibodies (E-cadherin, N-cadherin, vimentin, Snail, Slug, Histone H1, ERK, p-ERK, NF-κB-p50, p65or beta-actin).

The bands were measured with an enhanced chemiluminescence kit (Millipore, Billerica, MA, USA) and exposed by autoradiography. The densitometric analysis was carried out with Image J software (GE Healthcare, Buckinghamshire, UK).

### Statistical analysis

All data are presented as the mean±standard deviation. Differences between groups were assessed using the Student's t-test followed by the Shapiro-Wilk W test or one-way analysis of variance followed by Bonferroni's test. P<0.05 was considered to indicate a statistically significant difference. Analyses were performed using SPSS 17.0 software (SPSS Inc., Chicago, IL, USA).

## Results

### The serum concentrations of IL-17A was related with the clinicopathological features of the GBC cases

Patients with lymph node metastasis have higher serum concentrations of IL-17A, compared with those without node metastasis. Patients with advanced stage (Ⅲ-Ⅳ) also have higher serum concentrations of IL-17A. The serum concentrations of IL-17A was associated node metastasis and advanced stages (Table 1).

### IL-17A increases cell motility in GBC cells

**Figure 1:**
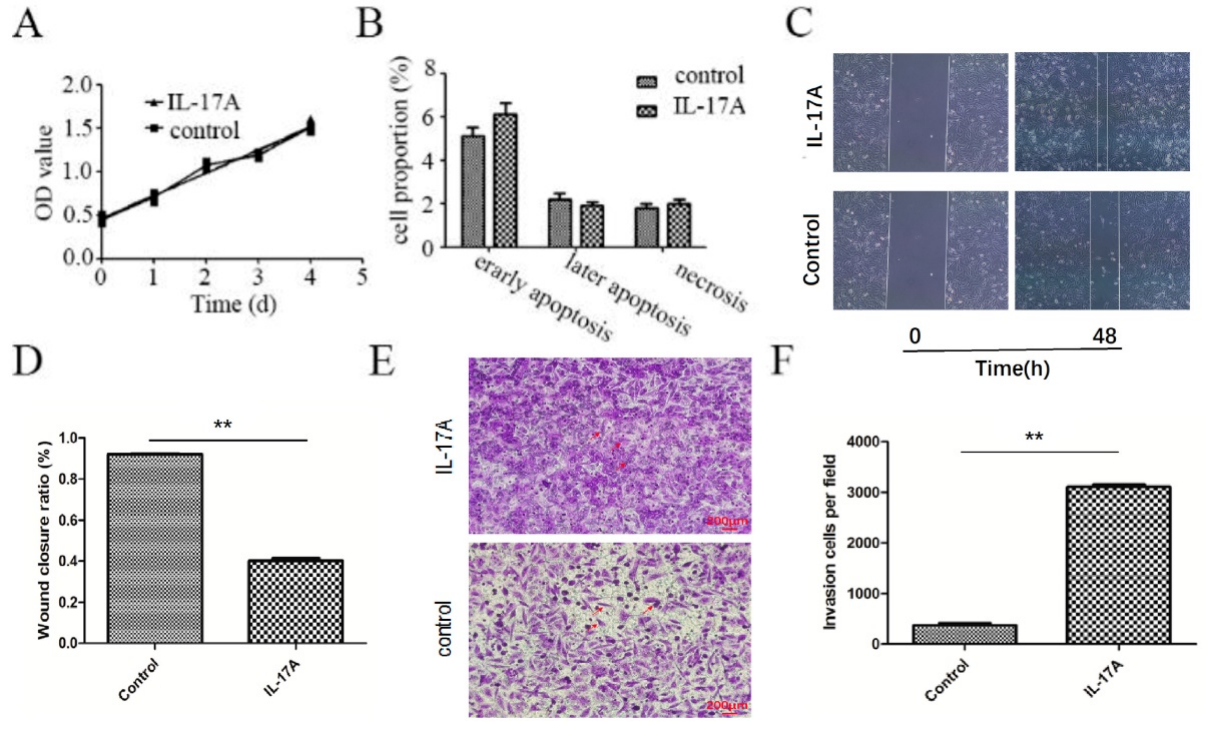
The pro-metastasis effect of IL-17A in GBC. (A) IL-17A has no effect on the proliferation of GBC cells. (B) IL-17A has no effect on the apoptosis of GBC cells. (C and D) The effect of IL-17A on cell migration was detected by using wound healing assays. IL-17A enhanced the motility of GBC cells. (E) The pro-invasion effect of IL-17A on GBC cells was assay via transwell assay. (F) The invasion cells per field were calculated. All data are presented as the mean±standard deviation. *p < 0.05 was considered to indicate a statistically significant difference.

We found that IL-17A had no effect on the apoptosis and proliferation of GBC-SD cells (Figure 1A and 1B). As indicated in Figure 1C and 1D, the wound closure areas in the IL-17A groups were large, respectively. The pro-migration effect of IL-17A was also verified using chambers with matrigel. Treatment with IL-17A markedly enhanced the migration ability of cells. Furthermore, as shown in Figure 1E and 1F, IL-17A increased the number of cells that pass through the membrane significantly, suggesting the pro-invasion effect of IL-17A. In conclusion, IL-17A could enhance the ability of migration and invasion of GBC cells.

### IL-17A activates the activity of ERK signal pathway in GBC cells

**Figure 2:**
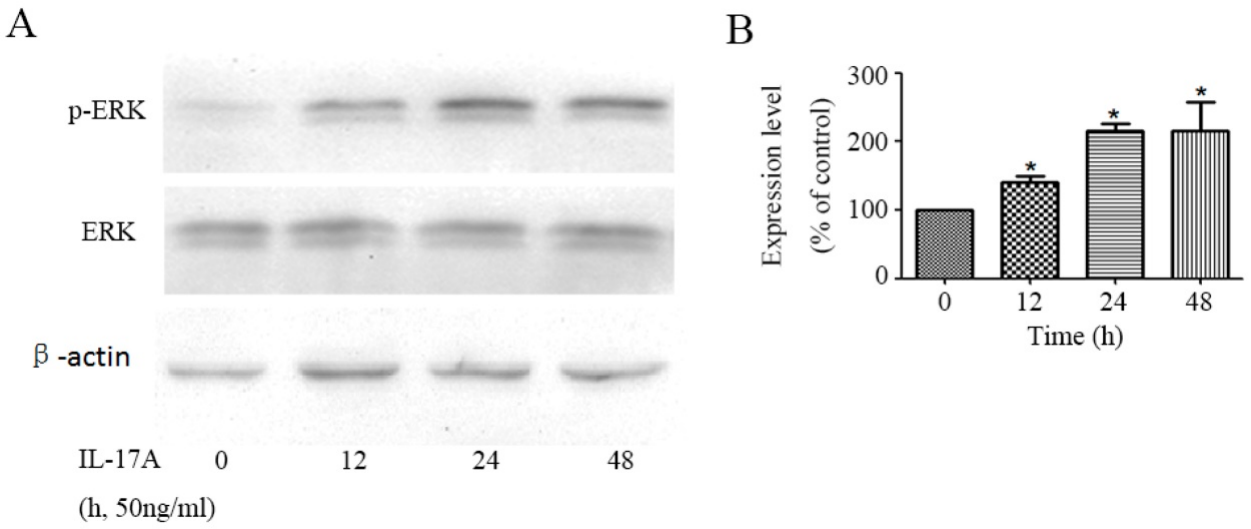
IL-17A activates ERK signal pathway in GBC cells. (A)The protein level of p-ERK and ERK was analysed with Western blotting analysis in GBC cells treated with IL-17A (50 ng/ml) at different time points. (B) Phosphorylation densities of ERK were digitally scanned. All data are presented as the mean±standard deviation. *p < 0.05 was considered to indicate a statistically significant difference.

We detected the effect of IL-17A on the activity of ERK signal pathway in GBC cells. The phosphorylation of ERK1/2, which represents the activity of ERK signal pathway, was significantly up-regulated by IL-17A in a time depend manner (Figure 2). To further verify the specific effect of baicalein on ERK pathway, we transfected GBC cells with a plasmid (pcDNA3.1(+)-MEK1) expressing human MEK1 (Figure S1) and found that MEK1 enhanced the pro-invasion effect of IL-17A on cell invasion (Figure S2).

### IL-17A regulates EMT marker and related transcription factor expressions in GBC cells

**Figure 3:**
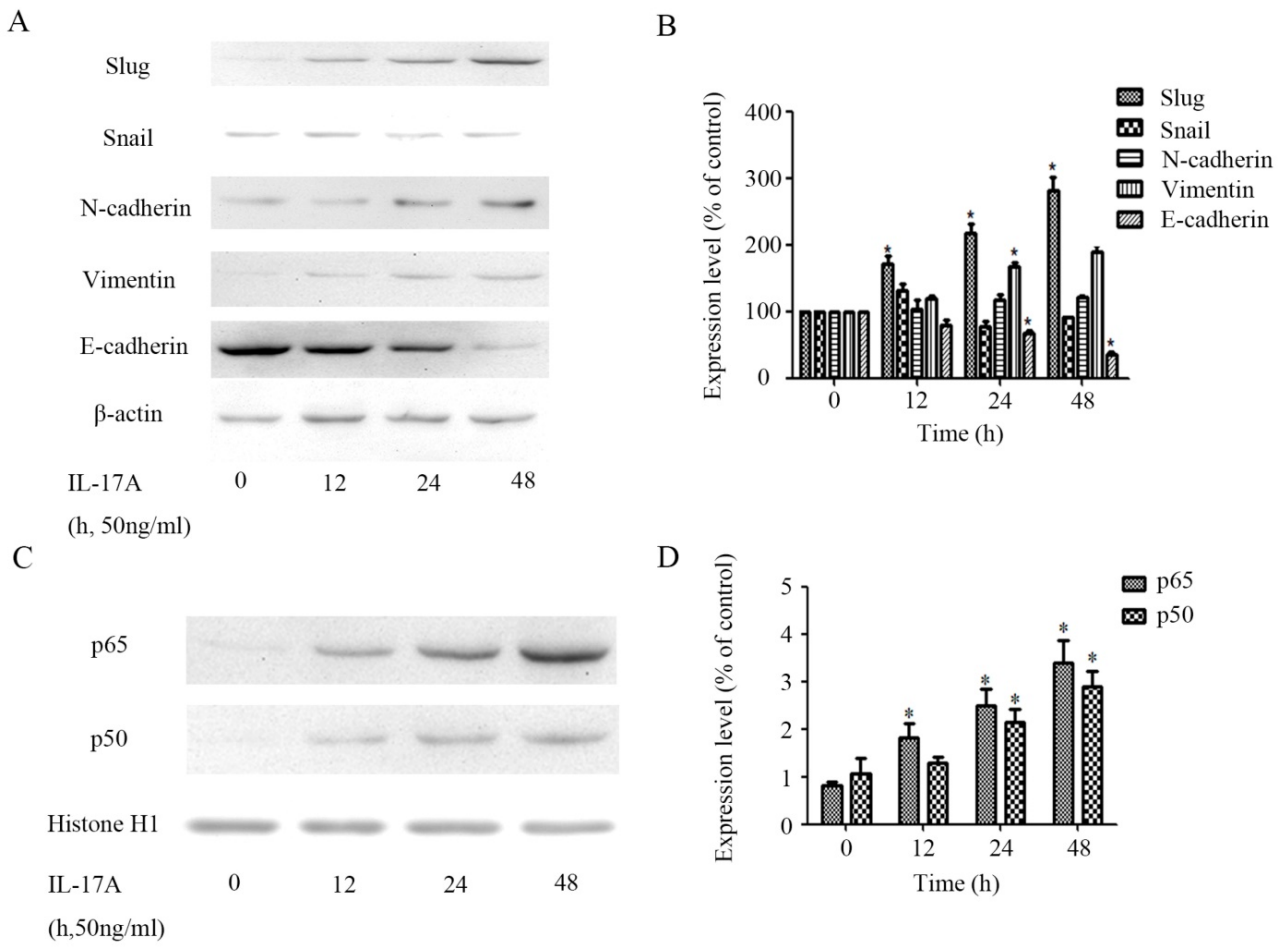
IL-17A induces EMT of GBC cells. (A) Protein levels of Snail, E-cadherin, N-cadherin, Vimentin and Slug in GBC cells treated with IL-17A (50 ng/ml) for 24 h or not were detected with western blot. (B) The expression levels of E-cadherin, Vimentin, N-cadherin, Snail and Slug were quantified as percentage of control. (C) The effect of IL-17A on protein expressions of NF-κB/p50 and p65. (D) Quantification of the expressions of NF-κB/p50 and p65. All data are presented as the mean±standard deviation. *p < 0.05 was considered to indicate a statistically significant difference.

The expression of epithelial marker E-cadherin in IL-17A treated group cells was significantly down-regulated (Figure 3A and 3B), while the expression of the mesenchymal phenotype markers vimentin was significantly increased. The expression of transcription factor slug can also be up-regulated by IL-17A (Figure 3A and 3B).

### IL-17A promotes the nuclear translocation of NF-κB

To further clarify the role of NF-κB transactivation in the pro-invasion effect of IL-17A, we detect the expression levels of transcription factors in GBC cells treated with IL-17A. We found that IL-17A significantly induced the nuclear translocation of NF-κB/ p65 and p50 (Figure 3C and 3D).

### IL-17A regulates EMT via activating ERK signal pathway

**Figure 4:**
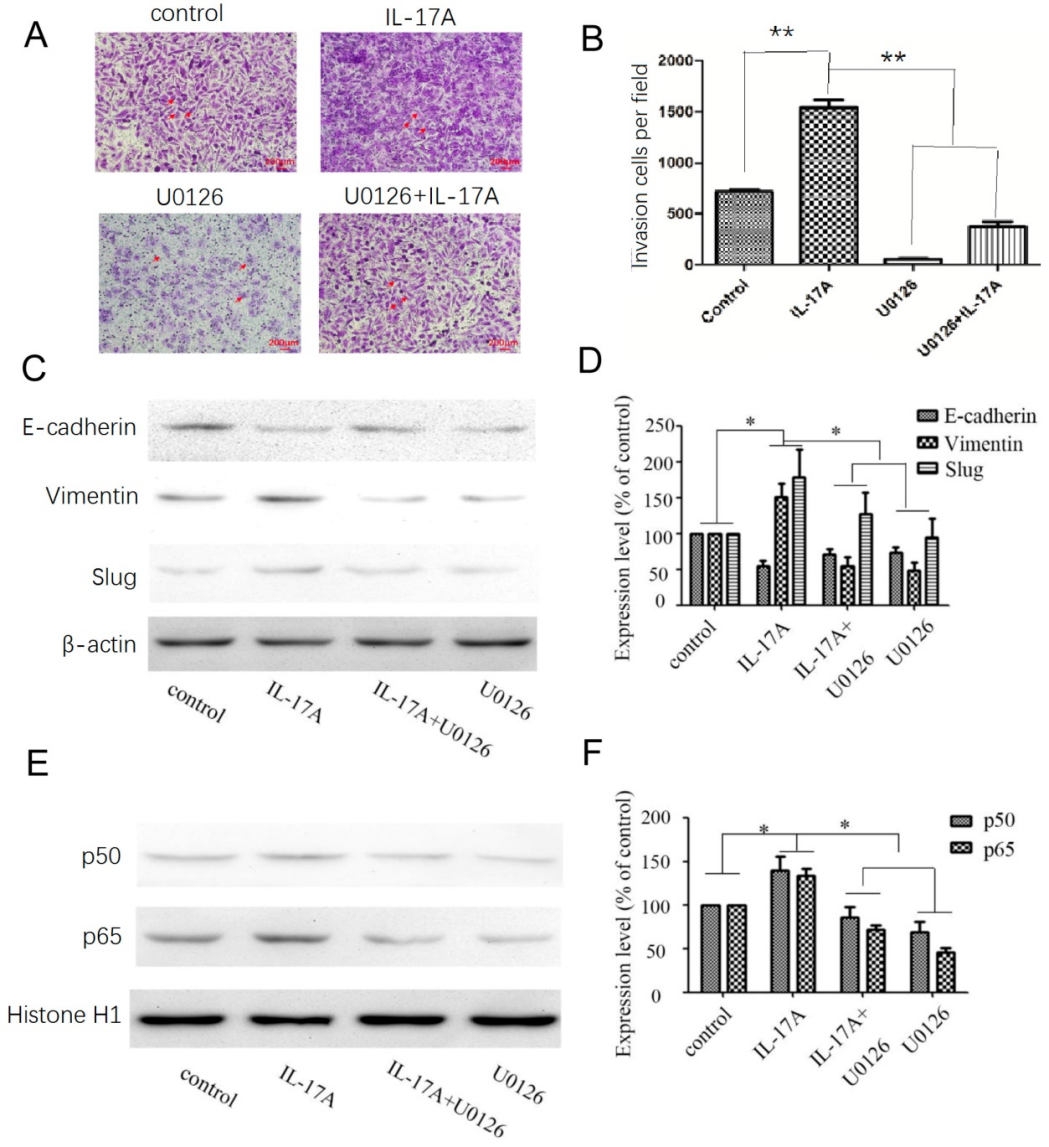
Effects of the ERK signal pathway inhibitor and IL-17A on cell invasion and EMT in GBC cells. (A, B) The pro-invasion effect of IL-17A in GBC cells could been partially reversed by U0126. (C, D) After treated with an ERK inhibitor (U0126; 10 μM) for 30 min, GBC cells were cultured with IL-17A or not. Pretreated with U0126 (10 μM), the expression levels of Vimentin were significantly decreased, the expression levels of E-cadherin were significantly increased. (E, F) Pretreated with U0126 could block the nuclear translocation of NF-κB/p50 and p65 in GBC cells. All data are presented as the mean±standard deviation. *p < 0.05 was considered to indicate a statistically significant difference.

After treated with an ERK inhibitor (U0126; 10 μM) for 30 min, GBC cells were cultured with IL-17A or not to explore the potential relationships between ERK signal pathway, NF-κB and EMT markers. Pretreated with U0126 (10 μM), the expression levels of vimentin were significantly decreased, the expression levels of E-cadherin were significantly increased (Figure 4C-D) compared with IL-17A treated cells. The study clarified that IL-17A induced EMT in GBC cell via activating ERK signal pathway. Pretreated with U0126 could block the nuclear translocation of NF-κB/p50 and p65. Accordingly, the pro-invasion effect of IL-17A in GBC cells could also been partially reversed by U0126 (Figure 4A and B). In conclusion, our results show that IL-17A up-regulates the activity of ERK signal pathway, then induces the nuclear translocation of NF-κB/p50 and p65, subsequently induces EMT, and promotes the pro-invasion effect in GBC cells at last.

## Discussion

Relationship between inflammation and tumorigenesis is well known, chronic infection has been found to be associated with GBC formation, but the contribution is still unclear and requires more investigation 9. Because of its aggressive factor, GBC is usually diagnosed at terminal stages, the prognosis is poor 10. In our previous study, we found a pro-invasion effect of IL-17A in esophageal adenocarcinoma cells 5. Our results show that IL-17A enhanced the invasion of GBC cells without effecting the proliferation and apoptosis. IL-17A up-regulated the activity of ERK signal pathway, then promoted the nuclear translocation of NF-κB and promoted EMT at last.

GBC is related with chronic inflammatory diseases closely, in which various inflammatory cytokines are detected. IL-17A plays crucial roles in the occurrence and development of lots of inflammatory diseases and is also frequently found in cancer immunity and microenvironment 5, 11. In the study, we clarified that the serum levels of IL-17A were associated with lymphatic metastasis and advanced stages. The results were similar to previous research, in which IL17 producing TCRγδ (+) in peripheral blood was related to poor prognosis of GBC patients^12^, IL-17+ cells in the tumor cooperatively facilitated pathogenesis and progression of GBC 13. But all the results are from retrospective clinical study, the molecular mechanisms of IL-17 in the development and prognosis of GBC need to be further explored.

Our results showed the pro-invasion effect of IL-17A in GBC cells. EMT could enhance the ability of movement and deformation of cancer cells, further cause the spread of cancer cells. Acquisition of mesenchymal markers, loss of epithelial markers and dysfunction of EMT-related transcription factors have been detected and are related with the prognosis and clinicopathology of GBC patients 11. The reduction of the protein level of E-cadherin is related with loss of adhesive mechanisms of GBC and CD8+ T cell infiltration at invasive areas of GBC 14, 15. Compare with primary tumors, the expression of vimentin is higher in metastases and is related with GBC lymphatic metastasis and metastasis 16. Slug, a EMT-related transcription factor, is one of the events that influence the regulation of EMT in GBC cells 17. To further exploit the mechanism of the pro-invasion effect of IL-17A on GBC cell, we detected the effect of IL-17A on cell EMT. In the study, we found that IL-17A could significantly up-regulate the protein level of vimentin and slug, and down-regulated the protein level of E-cadherin. Therefore, the pro-invasion effect of IL-17A on GBC is associated with inducing EMT.

Accumulating evidence suggests the cooperation of signal pathways take part in the stepwise EMT regulatory network in GBC, including ERK 18 and NF-κB 19. The over-activity of ERK signal pathway is found in both GBC tissues and cell lines 20. As a key regulator of the invasion of gallbladder cancer 21, IL-17A exerts its effect via regulating NF-κB in various cell lines 5. So we investigated whether the induction of EMT by IL-17A is via regulating the ERK and NF-κB signal pathway. In the study, we found that IL-17A enhanced the activity of ERK signal pathway and subsequently nuclear translocation of the p65 and p50 subunits of GBC cells. The pro-invasion effect could be partly reversed by ERK inhibitor, suggesting that ERK/NF-κB signal pathway played an important role in the induction of EMT by IL-17A on GBC cells. In conclusion, our results showed that IL-17A could enhance the ability of migration and invasion of GBC cells via inducing EMT via regulating ERK/NF-κB signal pathway. Further characterization of the pro-invasion effect of IL-17A may help us to find therapeutic targets and diagnostic marker in future.

## Supplementary Material

Supplementary figures.

## Figures and Tables

**Figure 5:**
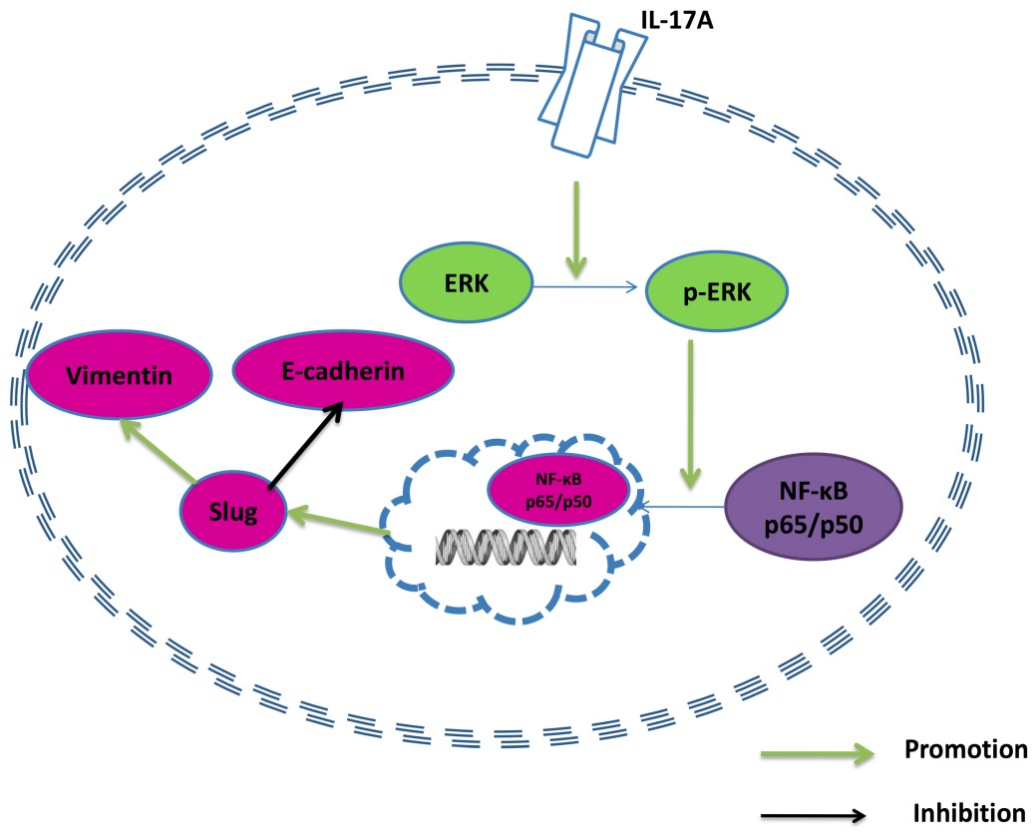
A model of the effect of IL-17A in GBC cells.

**Table 1:** Association between Serum levels of IL-17A and the clinicopathological features of the GBC cases.

Variables	No. of cases	Serum levels of IL-17A (pg/ml)	P-value
**Age (years)**			0.981
<60	10	7.4020	
≥60	24	7.4554	
**Sex**			0.804
Female	17	7.6894	
Male	17	7.1900	
**Histological grade**			
Well and moderate	24	7.1283	0.631
Poor	10	8.1870	
**N status**			
N0	21	5.7395	0.025
N1/2	13	10.162	
**Tumor size (cm)**			0.803
<5	23	7.6126	
≥5	11	7.0782	
**Clinical stage**			0.004
Ⅰ-Ⅱ	9	2.9689	
Ⅲ-Ⅳ	25	9.0492	
