# Supplementary material for: Interleukin 17A promotes gallbladder cancer invasiveness via ERK/NF-κB signal pathway mediated epithelial-to-mesenchymal transition
Source: J Cancer. 2020 May 18;11(15):4406–12. doi: 10.7150/jca.40656 (PMC7255371; doi:10.7150/jca.40656)

Figure S1 After transfecting with pcDNA3.1(+)-MEK1, the activity of ERK increased. Western blot analysis was performed to detect the phosphorylation level of ERK1/2 in GBC cells. All data are presented as the mean±standard deviation. ***p* < 0.01, **p* < 0.05 compared with the control or negative group.

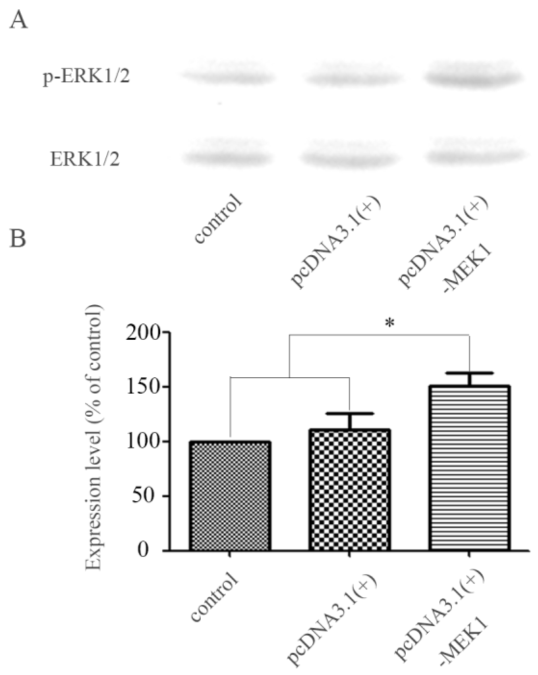

Figure S2 The over-activity of ERK signal pathway enhanced the pro-metastatic effect of IL-17A.

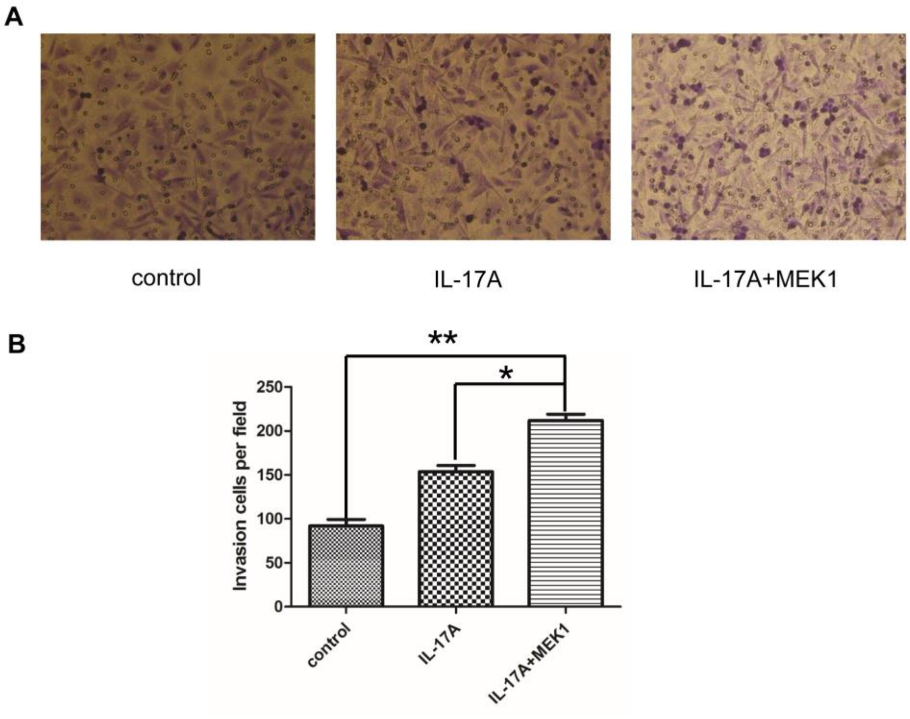

Supplement: Supplementary file 1 — Supplementary figures. [file jcav11p4406s1.pdf]
